# Polybrominated Diphenyl Ethers (PBDEs) and Hexabromocyclodecane (HBCD) in Composite U.S. Food Samples

**DOI:** 10.1289/ehp.0901345

**Published:** 2009-10-28

**Authors:** Arnold Schecter, Darrah Haffner, Justin Colacino, Keyur Patel, Olaf Päpke, Matthias Opel, Linda Birnbaum

**Affiliations:** 1 University of Texas School of Public Health, Dallas, Texas, USA; 2 University of Texas Southwestern Medical School, Dallas, Texas, USA; 3 University of Michigan School of Public Health, Ann Arbor, Michigan, USA; 4 Eurofins, Hamburg, Germany; 5 National Institute of Environmental Health Sciences, National Institutes of Health, Department of Health and Human Services, Research Triangle Park, North Carolina, USA

**Keywords:** dietary intake, food, HBCD, PBDE, United States

## Abstract

**Objectives:**

This study was designed to update previous U.S. market basket surveys of levels and polybrominated diphenyl ether (PBDE) dietary intake calculations. This study also quantifies hexabromocyclododecane (HBCD) levels in U.S.-purchased foods for the first time and estimates U.S. dietary intake of HBCD. This is part of a larger market basket study reported in two companion articles, of current levels of certain persistent organic pollutants (POPs) PBDEs, HBCD, perfluorinated compounds, polychlorinated biphenyls, and pesticides in composite food samples collected in 2008–2009.

**Methods:**

In this study, we measured concentrations of 24 PBDE congeners and total HBCD in composite samples of 31 food types (310 samples). U.S. dietary intake of PBDEs and HBCD was estimated referencing the most current U.S. Department of Agriculture loss-adjusted food availability report.

**Results:**

Total PBDE concentrations in food varied by food type, ranging from 12 pg/g wet weight (ww) in whole milk to 1,545 pg/g ww in canned sardines and 6,211 pg/g ww in butter. Total HBCD concentrations also varied substantially within and among food groups, ranging from 23 pg/g in canned beef chili to 593 pg/g in canned sardines. HBCD was not detected in any dairy samples. Dietary intake of all PBDE congeners measured was estimated to be 50 ng/day, mostly from dairy consumption but also from meat and fish. HBCD intake was estimated at 16 ng/day, primarily from meat consumption.

**Conclusion:**

PBDEs and HBCDs currently contaminate some food purchased in the United States, although PBDE intake estimated in this study is lower than reported in our previous market basket surveys. HBCD is in food at higher levels than expected based on previously reported levels in milk and blood compared with PBDE levels and is comparable to European levels.

Polybrominated diphenyl ethers (PBDEs) are bioaccumulative brominated flame retardants currently ubiquitous in the environment (Hites 2004; Hoh and Hites 2005). They are additives in some plastics, foams, electronics, and fabrics, originating from three commercial mixtures: penta-, octa-, and deca-BDE. Hexabromocyclodecanes (HBCDs) are also brominated flame retardants readily found in the environment (de Wit 2002). HBCDs are frequently used in polystyrene foams in furniture or building insulation and can be found in electrical equipment (Alaee et al. 2003). They can be used as PBDE substitutes (Covaci et al. 2006).

In the United States, penta- and octa-BDE production has been phased out. Washington and Maine are the first U.S. states to regulate deca-BDE (Betts 2008). The European Union has banned penta-BDE and octa-BDE products and has begun controlling deca-BDE. Currently there are no barriers to HBCD use or production in the United States (Covaci et al. 2006). Because of their persistence and resistance to degradation, PBDEs should remain in the environment for a long time, as occurred with the structurally similar polychlorinated biphenyls (PCBs). Although PCB levels have declined in the environment and in humans, PBDE concentrations have markedly increased in humans over the past decades (Doucet et al. 2009; Norén and Meironyté 2000; Schecter et al. 2005).

PBDEs have been detected in human milk (Schecter et al. 2003, 2005), blood (Mazdai et al. 2003; Schecter et al. 2005), adipose tissue (Fernandez et al. 2007; Guvenius et al. 2001; Johnson-Restrepo et al. 2005), and fetal liver (Schecter et al. 2007). Epidemiologic studies reported an association between PBDE exposure and decreased thyroid function (Herbstman et al. 2008), impaired spermatogenesis (Akutsu et al. 2008), and endocrine disruption (Darnerud 2008; Meeker et al. 2009). Limited studies of HBCDs in humans have reported detectable levels in milk, blood, and adipose tissue (Covaci et al. 2006; Eljarrat et al. 2009; Johnson-Restrepo et al. 2008; Ryan et al. 2006; Weiss et al. 2004). Few studies have examined human health effects of HBCDs. Rodent models have shown effects on neurotransmitter levels (Mariussen and Fonnum 2003), neurobehavioral function (Lilienthal et al. 2009), carcinogenesis (Ronisz et al. 2004), thyroid dysfunction (Darnerud 2003; Yamada-Okabe et al. 2005), and endocrine disruption (Darnerud 2003; Legler 2008; Yamada-Okabe et al. 2005).

Because depot sources of PBDEs remain in the environment, they continue to contaminate both food and dust, major sources of human exposure to PBDEs (Birnbaum and Staskal 2004; Jones-Otazo et al. 2005; Lorber 2007; Wu et al. 2007). Previous studies have attempted to quantify PBDE food concentrations and dietary intake (Huwe and Larsen 2005; Schecter et al. 2006). They concluded that the highest U.S. dietary PBDE exposure results from meat consumption. Dairy and fish also contributed to U.S. PBDE dietary intake. Overall, PBDE levels in food are higher in the United States than in European and Asian countries (Darnerud et al. 2006; Gomara et al. 2006; Schecter et al. 2006; Voorspoels et al. 2007).

In addition to describing HBCD in U.S. food for the first time, in this study we reevaluate and update PBDE levels in foodstuffs purchased in the United States in items previously examined and include some additional common foods. Some studies have examined the levels of HBCD in European food (Knutsen et al. 2008; van Leeuwen and de Boer 2008; van Leeuwen et al. 2009). For the first time in our market basket studies, new U.S. Department of Agriculture (USDA) food intake estimates are used to provide a better estimate of daily dietary intake in the United States (USDA 2009).

## Materials and Methods

### Sample collection

Ten samples of 31 distinct food types (310 samples total) were collected from five supermarkets on two separate occasions in Dallas, Texas (USA), in 2009. The samples included meat products (ground beef, bacon, turkey, sausage, ham, chicken breast, roast beef, canned chili containing ground beef), fish (salmon, canned tuna, catfish, tilapia, cod, canned sardines in water, frozen fish sticks), dairy foods (butter, milk, cream cheese, ice cream, frozen yogurt, yogurt, American cheese, and other cheeses—mozzarella, Colby, cheddar, Swiss, provolone, and Monterrey jack), vegetable-based foods (olive oil, canola oil, margarine, cereal, apples, potatoes, peanut butter), and eggs. Perishable samples were frozen at −80°C before shipping on dry ice to a Eurofins laboratory in Hamburg, Germany. Equal weights of each of the 10 samples of the 31 food types were homogenized and combined into 31 composite samples to determine mean levels of contamination in these U.S.-purchased foods.

### Chemical analysis

The analytical methods for PBDE measurement in food have been described previously (Päpke et al. 2004; Schecter et al. 2004). We used a modification that employed a shorter, 12-m column and negative ion methodology for improved octa- through deca-BDE measurements.

For HBCD, three native standards [α-, β-, γ-HBCD (mixture 1:1:1)] and two internal ^13^C_12_-labeled standards (γ-HBCD, BDE-138) were obtained from Wellington Laboratories (Guelph, Ontario, Canada). Extraction was done identically to PBDE procedure after adding ^13^C_12_-labeled γ-HBCD to the samples. Cleanup of lipid extracts was performed by acid-treated alumina oxide columns. The final extract was reduced to a volume of 50 μL by a stream of nitrogen containing ^13^C_12_-labeled BDE-138 for recovery standard. Measurements were performed using gas chromatography/mass spectrometry with negative chemical ionization mode and DB 5 (15 m, 0.25 mm inner diameter, 0.1 μm film) column for gas chromatographic separation. Identification of HBCD (as the sum of stereoisomers α, β, and γ) was based on retention time and isotope ratio.

### Dietary intake estimation

To estimate dietary intake of PBDEs and HBCD, we used the 2007 USDA food availability data (USDA 2009). In the past, dietary intake has been calculated using the 1994–1996 USDA Continuing Survey of Food Intake by Individuals (Huwe and Larsen 2005; Schecter et al. 2004, 2006). Over the past 10 years, these data about child and adult food consumption have changed (Adair and Popkin 2005; Nielsen et al. 2002; Popkin and Gordon-Larsen 2004; Popkin et al. 2003). Calculating dietary intake estimate based on the 2007 USDA food availability data provides updated dietary estimates (USDA 2009). USDA provides loss-adjusted food availability data to represent daily food consumption of Americans, males and females, in grams of food consumed daily per capita over a lifetime. Concentrations of measured chemicals per food type from this study were multiplied by the USDA estimates.

Where concentrations of PBDEs and HBCD were below the limit of detection (LOD), concentrations were estimated as zero for dietary intake estimations. All detected PBDE congeners in each sample were added to obtain the total PBDE level. HBCD intake was also calculated from the one HBCD measurement. In both instances, we calculated nondetected values (NDs) as zero. We used the zero value (lower-bound estimate) instead of the half LOD in calculating dietary intake so as not to overestimate dietary intake. For most food samples analyzed, these two numbers were quite similar.

## Results

### PBDEs

Measured PBDE congeners are reported in Tables 1–4. Total PBDE levels were calculated as the sum of the reported congeners using zero for NDs.

The most heavily PBDE contaminated food was butter with total concentration of 6,180 pg/g wet weight (ww). The next highest contaminated items were canned sardines and fresh salmon, with 1,487 and 925 pg/g ww, respectively. High total PBDE concentration in butter was driven largely by BDE-209 (5,190 pg/g ww), BDE-207 (359 pg/g ww), and BDE-206 (224 pg/g ww). PBDEs in canned sardines (1,487 pg/g ww) consisted primarily of BDE‐47, BDE-49, BDE-99, and BDE‐100, with respective concentrations of 798, 259, 149, and 159 pg/g ww. The greatest contributors to salmon were from BDE-47 and BDE-49, with respective concentrations of 486 and 139 pg/g ww.

BDE-47 was the most prevalent PBDE congener, detected in 30 of 31 samples analyzed. The only sample with a concentration lower than the LOD was canola oil, possibly due to the relatively high LOD (2.02 pg/g ww) in this high-lipid-containing food. BDE-99 was the second most frequently detected congener, found in 28 of 31 food samples; concentrations were below the LOD in tilapia, canola oil, and peanut butter. LODs for canola oil and peanut butter were also relatively high, 5.46 and 4.58 pg/g ww, respectively, perhaps resulting in these high-lipid-content samples having concentrations below the LOD.

### HBCD

HBCD was present at the highest concentrations in canned sardines (593 pg/g), followed by fresh salmon (352 pg/g) and then peanut butter (300 pg/g). HBCD concentration was below the LOD in ground beef, canned tuna, cod, olive oil, canola oil, margarine, cereals, eggs, apples, potatoes, and dairy products. HBCD was detected in 13 of 31 samples: seven of eight meats, five of seven fish, no eggs or dairy products, and one of seven vegetables.

### Dietary intake

**Figure 1:**
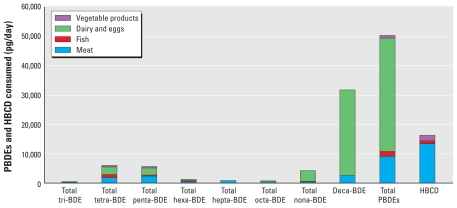
Estimation of per capita dietary exposure to PBDEs and HBCD using 2007 USDA food availability data (USDA 2009) for all ages. Samples where PBDE and HBCD concentrations were below the LOD were estimated as zero.

Samples below the LOD were estimated as zero. Figure 1 shows total daily intake estimated by this method. The largest dietary intake of PBDEs in this study was from dairy and eggs (38.6 ng/day), largely from BDE-209 in butter (28.7 ng/day). Meat accounted for the second highest PBDE intake (9.1 ng/day), primarily from beef and pork, specifically hamburger for beef and sausage and bacon for pork. Vegetable products accounted for 1.0 ng/day, mostly due to high LODs. Fish accounted for 1.6 ng/day. Total PBDE intake was estimated at 50.3 ng/day.

Total dietary intake of HBCD, with samples below the LOD estimated to zero, was 15.4 ng/day (Figure 1). The largest contribution to intake was from meat (12.5 ng/day), largely from pork (4.2 ng/day) and chicken (4.2 ng/day). HBCD intake from fish, vegetable products, and dairy and eggs was 0.9 ng/day, 2.0 ng/day, and 0 ng/day, respectively.

## Discussion

The findings from this part of our study confirm that some U.S. food remains contaminated with PBDEs and establish that HBCD can be found in certain commonly consumed foods. In this study, the highest total PBDE levels were measured in butter and fish samples, specifically salmon and canned sardines. Lowest detected levels were found in other dairy samples (whole milk and yogurt) and in vegetables (cereal, apples, and potatoes). Based on this study, the main route of dietary PBDE intake in the U.S. population appears to be from dairy, followed by meat products and then fish.

Previous surveys of PBDEs in U.S. food have established that there is considerable variability, even within the same food types (Huwe and Larsen 2005; Schecter et al. 2004, 2006). Although this study adds to and extends our previous market basket surveys as part of the most comprehensive measurement of PBDEs in U.S. food to date, the results cannot be considered as representative as that which could be obtained from a larger-scale but considerably more expensive sampling program, such as one resembling the USDA’s Pesticide Data Program (USDA 2008).

Compared with our previous market basket surveys, we found a comparable congener distribution, with BDE congeners 47, 99, 100, 153, 154, and 209 comprising the bulk of the total PBDE level when detected (Schecter et al. 2004, 2006). Similarly, BDE-99 was detected at higher levels than BDE-100 in most meat, dairy, and vegetable samples, which is consistent with the usual congener pattern of the DE-71 penta-BDE commercial mixture: approximately 48.6% BDE-99 and 13.1% BDE-100 (La Guardia et al. 2006).

BDE-153 was detected at higher levels than BDE-154 in meat and dairy but not in fish. Although BDEs 153 and 154 constitute nearly equal amounts of the DE-71 commercial mixture (5.4% and 4.5%, respectively), BDE-153 is found at higher levels in the DE-79 commercial mixture (8.7% vs. 1.1%). Previous studies have found BDE-154 in higher amounts than BDE-153 (Hayward et al. 2007). This high level of BDE-154 may be indicative of its metabolic stability (Hakk et al. 2009) and/or the metabolic conversion of BDE-183 to BDE-154 (Stapleton et al. 2004).

BDE-209 was detected at higher levels in certain dairy products, especially butter (5,190 pg/g ww) and cheese (161 pg/g ww). The reason for this elevation is unknown but may result from manufacturing and production introducing contaminants, possibly from wrapping material. The improved analytic methods used for BDE-209 led us to believe the results are valid, but contamination of some butter outliers may have produced atypically high values for the composite samples. Further studies will include analysis of individual butter samples. BDE-49 was also detected in relatively high concentrations in fish (salmon, 139 pg/g ww; canned sardines, 259 pg/g ww). Because BDE-49 is present in < 1% in both penta-BDE commercial mixtures (DE-71 and DE-79) and not in octa-BDE or deca-BDE commercial mixtures (La Guardia et al. 2006), its presence may well be attributable to breakdown of higher brominated congeners. Similarly, the high levels of nona-BDE congeners in butter (BDE-206, 224 pg/g ww; BDE-207, 359 pg/g ww) were possibly from BDE-209 debromination.

Comparing our previous market basket intake estimates of 88.5 ng/day to the 50.3 ng/day calculated in this study, dietary intake was lower in this study, possibly consistent with decreasing PBDE exposure through U.S.-purchased foods or with the use of zero for NDs. In the previous study, the largest portion of estimated dietary intake of PBDEs was due to meat consumption. In this study, dairy intake contributed the largest portion of estimated dietary PBDE intake, due to high levels of PBDEs measured in the butter composite sample.

It may be helpful to also estimate daily intake using half the LOD for comparison with past studies. Regardless of whether zero or half the LOD is used for samples with levels below the LOD, the resulting daily dietary intake still only represents intake for average Americans on the basis of per capita lifetime food availability estimates. Public health policy often aims to protect up to 95% of the population as opposed to merely the 50th percentile. These dietary estimates of PBDEs and HBCDs can serve as a guide to the general public’s exposure but may not be sufficiently protective for those with higher exposures to guide future policy and regulations.

Compared with other studies that measured PBDEs in food and estimated total dietary intake, results from this study were relatively similar when estimating total PBDE intake through the diet. A Belgium market basket survey measuring PBDEs in food estimated that the largest contribution to dietary PBDE intake was from fish (460 pg/g ww), rather than dairy consumption as found here (Voorspoels et al. 2007). Total PBDE exposure ranged from 28 to 35 ng/day. A Belgium duplicate diet study of university students was conducted as part of a larger study to determine which factors influence serum concentrations of PBDEs (Roosens et al. 2009a). Estimated average dietary exposure for tri-BDE through hepta-BDE congeners was 10 ng/day, whereas median estimated dietary exposure for BDE-209 was 95 ng/day (Roosens et al. 2009a). The BDE-209 median may overestimate the true value because of high LODs (43–169 pg/g ww) and estimating NDs as one-half the LOD. A Spanish study reported highest PBDE levels in fish (564 pg/g ww) (Domingo et al. 2008). High levels were also detected in fats and oils (359 pg/g ww), with NDs estimated as one-half the LOD. Total dietary intake of a standard adult male in Catalonia was estimated at 75 ng/day, a 23% decrease from a Spanish 2000 estimate (Domingo et al. 2008).

Because commercial penta-BDE and octa-BDE products are no longer being produced or used in the United States, and deca-BDE is also coming under scrutiny, attention has moved toward HBCD as a PBDE alternative. European studies from birds and eggs have suggested rising levels from as early as 1969 (Sellstrom et al. 2003). In California, HBCD levels in sea lions appeared to increase exponentially between 1993 and 2003, doubling every 2 years (Stapleton et al. 2006). HBCD concentrations were reported to be lower in North America than in Europe based on levels in fish, dolphins, sea lions, and air (Covaci et al. 2006).

Fish samples purchased in 2007 and 2008 in the Netherlands showed HBCD concentrations on the same order of magnitude or lower than those we measured in the present study (van Leeuwen et al. 2009). Dutch salmon contained HBCD concentrations of 100 compared with the 352 pg/g ww reported here. Compared with our ND levels of HBCD, this study detected 180 pg/g ww in tilapia. A different Dutch study reported two samples of farm-raised salmon at < 100 pg/g ww and 1,300 pg/g ww (van Leeuwen and de Boer 2008).

A 2008 Norwegian study performed an analysis of HBCDs in fish, meat, and dairy products and a dietary intake calculation (Knutsen et al. 2008). HBCD concentrations in farmed salmon ranged from 128 to 545 pg/g ww compared with 352 pg/g ww for salmon in our study. The Norwegian HBCD concentration range in sardines of 633–957 pg/g ww was similar to the 593 pg/g ww detected in the present study. For meat, the Norwegian value of 21 pg/g ww in pork was much lower than the value of 190 pg/g ww measured in bacon in the present study. Estimated Norwegian dietary intake of HBCD was 0.33 ng/kg/day, largely from oily fish (Knutsen et al. 2008). A recently published study measured levels of HBCD in 165 duplicate diet samples from Belgium (Roosens et al. 2009b). The estimated dietary intake of HBCD ranged from 1.2 to 20 ng/day and averaged 7.2 ng/day, with the bulk of HBCD detected in food being γ-HBCD. The estimated dietary intake for HBCD in the present study was 15.3 ng/day, which is higher than the average intake in the Belgian study, but within the estimated range.

It appears that U.S. intake of HBCD is comparable to estimates from Europe. Using dietary intake estimates from the USDA food availability data, American HBCD intake at 0.50 ng/kg/day for an individual weighing 70 kg is greater than Norwegian HBCD intake at 0.33 ng/kg/day. Unlike the Norwegian dietary intake of HBCD largely from oily fish (Knutsen et al. 2008), the greatest contribution to intake in this study was derived from meat.

The data we present here measuring PBDEs and HBCD, and those of the companion paper (Schecter et al. 2010) addressing perfluorinated compounds (PFCs), PCBs, and pesticides, in the same food samples using newer USDA dietary intake estimates extend and update previous work describing the contamination of U.S. food with a range of persistent organic pollutant (POP) contaminants. Estimated dietary intake of PBDEs in this study was lower than estimated in our previous market basket survey, and estimated dietary intake of HBCD was comparable to estimates from recent studies published by European research groups. However, the wide range of contaminants detected in these samples are still of some concern, because of the less than complete understanding of mechanisms of toxicity of these chemicals and the potential for additive or synergistic effects. Because of the unknown toxicity of mixtures of these emerging and classical POPs reported in this and the companion article (Schecter et al. 2010) reporting PFCs, PCBs, and pesticides from the same composite food samples, a larger representative sampling of food for a wide range of classical and emerging pollutants as well as increased food surveillance is strongly indicated. Because of the relative lack of data regarding dietary intake levels in humans, food HBCD levels, and health effects, it is important that exposure and health outcome research continue.

## Figures and Tables

**Table 1:** Levels of brominated flame retardants detected in composite meat samples (pg/g ww).

PBDE	Hamburger	Bacon	Sliced turkey	Sausages	Ham	Sliced chicken breast	Roast beef	Canned chili
Lipid percentage	21.70	36.10	2.00	23.90	4.30	4.70	4.60	9.10
Tri-BDE (BDE-17)	ND (0.18)	ND (0.05)	ND (0.04)	ND (0.19)	ND (0.09)	ND (0.17)	ND (0.09)	ND (0.07)
Tri-BDE (BDE-28)	1.00	0.90	0.53	0.64	0.44	ND (0.17)	0.38	0.27
Tetra-BDE (BDE-47)	13.60	25.20	3.94	63.30	3.15	6.32	6.22	7.77
Tetra-BDE (BDE-49)	ND (0.41)	ND (0.17)	ND (0.15)	ND (0.36)	ND (0.34)	ND (0.10)	ND (0.17)	ND (0.18)
Tetra-BDE (BDE-66)	ND (0.38)	ND (0.15)	ND (0.13)	ND (0.31)	ND (0.29)	ND (0.09)	ND (0.15)	ND (0.16)
Penta-BDE (BDE-85)	ND (0.58)	ND (0.52)	ND (0.57)	1.79	ND (0.34)	ND (0.61)	ND (0.26)	ND (0.36)
Penta-BDE (BDE-99)	15.80	24.70	5.99	72.60	2.72	8.68	4.62	8.39
Penta-BDE (BDE-100)	2.29	3.86	1.56	11.30	0.48	1.29	0.71	1.11
Penta-BDE (BDE-119)	ND (0.57)	ND (0.44)	ND (0.48)	ND (0.49)	ND (0.28)	ND (0.51)	ND (0.21)	ND (0.30)
Penta-BDE (BDE-126)	ND (0.39)	ND (0.30)	ND (0.32)	ND (0.34)	ND (0.19)	ND (0.34)	ND (0.14)	ND (0.20)
Hexa-BDE (BDE-138)	ND (0.61)	0.81	ND (0.36)	1.80	ND (0.34)	ND (0.29)	ND (0.32)	ND (0.25)
Hexa-BDE (BDE-153)	5.20	5.33	1.83	14.90	0.76	1.01	0.74	1.78
Hexa-BDE (BDE-154)	2.15	1.97	0.91	7.56	0.40	0.72	ND (0.21)	0.68
Hexa-BDE (BDE-156)	ND (0.83)	ND (0.48)	ND (0.58)	ND (0.95)	ND (0.55)	ND (0.47)	ND (0.52)	ND (0.41)
Hepta-BDE (BDE-183)	ND (1.57)	49.60	1.89	9.73	ND (0.75)	0.90	1.06	ND (0.50)
Octa-BDE (BDE-196)	ND (1.57)	5.73	ND (1.01)	2.69	ND (1.09)	ND (1.13)	ND (0.68)	ND (1.20)
Octa-BDE (BDE-197)	ND (2.23)	21.30	ND (1.01)	7.40	ND (1.09)	ND (1.13)	ND (0.61)	ND (1.08)
Nona-BDE (BDE-206)	ND (1.57)	2.48	1.42	ND (1.73)	ND (1.09)	ND (1.13)	ND (1.17)	ND (1.58)
Nona-BDE (BDE-207)	1.74	13.10	2.54	3.73	ND (1.09)	2.12	ND (1.17)	ND (1.37)
Deca-BDE (BDE-209)	ND (10.5)	36.80	39.40	10.00	ND (5.43)	33.70	ND (5.84)	8.90
Total PBDE lower bound	41.8	191.8	60.0	207.4	8.0	54.7	13.7	28.9
Total PBDE middle bound	52.5	192.8	62.3	209.6	14.4	57.8	19.5	32.7
HBCD	ND (60)	192	124	151	24	98	188	23

Lower bound indicates ND estimated as 0; middle bound indicates ND estimated as one-half the LOD. Numbers in parentheses denote LOD.

**Table 2:** Levels of brominated flame retardants detected in composite fish samples (pg/g ww).

PBDE	Salmon	Canned tuna	Catfish fillet	Tilapia	Cod	Canned sardines	Frozen fish sticks
Lipid percentage	11.90	14.80	11.60	1.60	0.30	10.30	10.30
Tri-BDE (BDE-17)	8.45	ND (0.16)	2.08	ND (0.23)	ND (0.1)	2.26	ND (0.36)
Tri-BDE (BDE-28)	28.00	ND (0.16)	2.50	0.90	0.59	40.20	ND (0.36)
Tetra-BDE (BDE-47)	486	9.01	79.40	7.70	18.00	798	11.50
Tetra-BDE (BDE-49)	139	2.67	12.00	2.25	1.54	259	ND (0.80)
Tetra-BDE (BDE-66)	15.40	ND (0.55)	ND (0.56)	ND (0.36)	ND (0.36)	18.70	ND (0.69)
Penta-BDE (BDE-85)	ND (0.88)	ND (0.47)	1.72	ND (1.04)	ND (0.50)	ND (1.66)	ND (1.03)
Penta-BDE (BDE-99)	69.70	1.98	54.70	ND (0.75)	1.05	149	2.67
Penta-BDE (BDE-100)	95.70	3.13	18.20	1.24	3.06	159	1.09
Penta-BDE (BDE-119)	9.61	ND (0.393)	ND (0.45)	ND (0.87)	ND (0.42)	5.50	ND (0.86)
Hexa-BDE (BDE-138)	1.63	ND (0.89 )	1.29	ND (0.68)	ND (1.15)	ND (0.60)	ND (1.24)
Hexa-BDE (BDE-153)	15.90	1.00	7.03	1.41	ND (1.03)	13.70	ND (1.12)
Hexa-BDE (BDE-154)	48.80	1.92	6.41	3.08	ND (0.77)	41.20	ND (0.83)
Hepta-BDE (BDE-183)	ND (1.66)	ND (1.30)	ND (2.62)	ND (0.83)	ND (0.67)	ND (1.48)	3.94
Octa-BDE (BDE-196)	ND (1.30)	ND (1.07)	ND (1.13)	ND (0.83)	ND (0.44)	ND (2.75)	ND (1.77)
Octa-BDE (BDE-197)	ND (1.30)	ND (1.07)	1.26	ND (0.83)	ND (0.44)	ND (2.60)	ND (1.77)
Nona-BDE (BDE-206)	ND (2.89)	ND (1.09)	3.01	ND (0.83)	ND (0.87)	ND (12.6)	ND (1.77)
Nona-BDE (BDE-207)	ND (2.50)	ND (1.07)	4.65	ND (0.83)	ND (1.29)	ND (8.93)	ND (1.77)
Deca-BDE (BDE-209)	7.19	ND (15.7)	50.80	ND (7.24)	ND (6.99)	ND (79.3)	ND (8.86)
Total PBDE lower bound	925.4	19.7	245.1	16.6	24.2	1486.6	19.2
Total PBDE middle bound	930.7	31.7	247.4	24.7	31.8	1541.5	30.8
HBCD	352.0	ND (29)	133	179.6	ND (59)	593	113.

Lower bound indicates ND estimated as 0; middle bound indicates ND estimated as one-half the LOD. Numbers in parentheses denote LODs.

**Table 3:** Levels of brominated flame retardants detected in composite dairy and egg samples (pg/g ww).

PBDE	Butter	American cheese	Other cheese	Whole milk	Ice cream	Frozen yogurt	Cream cheese	Whole milk yogurt	Eggs
Lipid percentage	91.4	25.3	30.1	3.8	16.2	3.1	34.0	2.9	10.0
Tri-BDE (BDE-17)	ND (1.29)	ND (0.33)	ND (0.29)	ND (0.03)	ND (0.17)	ND (0.22)	ND (0.12)	ND (0.09)	ND (0.11)
Tri-BDE (BDE-28)	5.29	0.37	0.98	ND (0.03)	0.28	0.32	0.37	ND (0.05)	ND (0.09)
Tetra-BDE (BDE-47)	173.00	20.20	53.10	2.57	23.60	2.57	42.20	2.08	8.71
Tetra-BDE (BDE-49)	ND (3.04)	ND (0.33)	ND (0.52)	ND (0.14)	ND (0.27)	ND (0.14)	ND (0.34)	ND (0.12)	ND (0.17)
Tetra-BDE (BDE-66)	ND (2.62)	ND (0.32)	ND (0.52)	ND (0.12)	ND (0.26)	ND (0.14)	ND (0.33)	ND (0.11)	ND (0.15)
Penta-BDE (BDE-85)	ND (5.16)	1.22	ND (1.19)	ND (0.11)	ND (0.47)	ND (0.16)	ND (0.38)	ND (0.24)	ND (0.39)
Penta-BDE (BDE-99)	157.00	14.10	37.00	1.93	18.40	1.38	26.50	1.35	18.50
Penta-BDE (BDE-100)	23.70	2.34	5.78	0.46	3.57	0.30	5.45	ND (0.16)	7.25
Penta-BDE (BDE-119)	ND (4.29)	ND (0.54)	ND (1.17)	ND (0.11)	ND (0.47)	ND (0.15)	ND (0.37)	ND (0.24)	ND (0.38)
Hexa-BDE (BDE-138)	ND (6.27)	ND (1.10)	ND (0.57)	ND (0.24)	ND (0.49)	ND (0.36)	ND (0.58)	ND (0.40)	ND (0.40)
Hexa-BDE (BDE-153)	16.80	2.99	6.37	0.42	2.48	ND (0.35)	4.07	ND (0.38)	7.26
Hexa-BDE (BDE-154)	5.40	1.33	2.53	ND (0.18)	1.30	ND (0.29)	2.00	ND (0.30)	3.59
Hepta-BDE (BDE-183)	ND (7.68)	1.24	ND (1.31)	ND (0.55)	ND (0.73)	ND (1.84)	ND (2.63)	ND (0.46)	ND (0.76)
Octa-BDE (BDE-196)	14.33	ND (1.11)	ND (1.21)	ND (0.17)	ND (0.90)	ND (0.79)	ND (0.93)	ND (0.37)	0.63
Octa-BDE (BDE-197)	11.43	ND (1.75)	ND (1.31)	ND (0.17)	ND (0.90)	ND (0.75)	ND (0.95)	ND (0.37)	ND (0.44)
Nona-BDE (BDE-206)	224.00	ND (2.22)	4.79	ND (0.40)	ND (2.84)	ND (2.09)	ND (1.86)	ND (0.85)	4.72
Nona-BDE (BDE-207)	358.67	ND (2.22)	5.30	ND (0.35)	ND (2.46)	ND (1.81)	ND (1.86)	ND (0.74)	2.88
Deca-BDE (BDE-209)	5190.00	ND (11.1)	161	4.46	17.6	ND (7.45)	16.00	5.61	34.3
Total PBDE lower bound	6179.6	43.8	276.9	9.8	67.2	4.6	96.6	9.0	87.8
Total PBDE middle bound	6194.8	54.3	280.9	11.1	72.2	11.9	100.5	11.5	89.3
HBCD	ND (128)	ND (4)	ND (4)	ND (17)	ND (15)	ND (20)	ND (57)	ND (16)	ND (11)

Lower bound indicates ND estimated as 0; middle bound indicates ND estimated as one-half the LOD. Numbers in parentheses denote LODs.

**Table 4:** Levels of brominated flame retardants detected in composite vegetable-based samples (pg/g ww).

PBDE	Olive oil	Canola oil	Margarine	Cereals	Apples	Potatoes	Peanut butter
Lipid percentage	100	100	79.4	0	0	0	50.5
Tri-BDE (BDE-17)	ND (2.17)	ND (1.97)	ND (2.52)	ND (0.34)	0.04	ND (0.12)	ND (1.52)
Tri-BDE (BDE-28)	ND (2.14)	ND (1.93)	ND (2.48)	2.62	0.06	ND (0.115)	ND (1.49)
Tetra-BDE (BDE-47)	15.30	ND (2.02)	15.30	0.58	5.61	2.89	ND (4.10)
Tetra-BDE (BDE-49)	ND (3.38)	ND (3.82)	ND (2.89)	ND (0.59)	0.23	ND (0.134)	ND (2.23)
Tetra-BDE (BDE-66)	ND (2.92)	ND (3.30)	ND (2.50)	ND (0.55)	ND (0.17)	ND (0.116)	ND (1.92)
Penta-BDE (BDE-85)	ND (4.52)	ND (7.59)	ND (3.88)	ND (0.73)	ND (0.22)	ND (0.346)	ND (6.37)
Penta-BDE (BDE-99)	9.87	ND (5.45)	28.60	1.61	5.22	2.63	ND (4.58)
Penta-BDE (BDE-100)	ND (2.64)	ND (4.43)	ND (2.27)	ND (0.50)	0.94	ND (0.202)	ND (3.72)
Penta-BDE (BDE-119)	ND (3.76)	ND (6.31)	ND (3.23)	ND (0.72)	ND (0.21)	ND (0.288)	ND (5.30)
Hexa-BDE (BDE-138)	ND (4.36)	ND (5.44)	ND (4.91)	ND (2.14)	ND (0.26)	ND (0.603)	ND (10.7)
Hexa-BDE (BDE-153)	ND (3.93)	ND (4.91)	ND (4.42)	ND (2.08)	0.43	ND (0.543)	ND (9.65)
Hexa-BDE (BDE-154)	ND (2.92)	ND (3.64)	ND (3.28)	ND (1.62)	ND (0.19)	ND (0.403)	ND (7.16)
Hepta-BDE (BDE-183)	ND (6.87)	ND (4.01)	ND (4.70)	ND (1.69)	ND (0.73)	ND (0.863)	ND (11.8)
Octa-BDE (BDE-196)	ND (6.45)	ND (6.84)	ND (5.71)	ND (1.97)	ND (0.43)	ND (0.55)	ND (5.12)
Octa-BDE (BDE-197)	ND (6.45)	ND (6.59)	ND (5.71)	ND (1.97)	ND (0.39)	ND (0.55)	ND (5.12)
Nona-BDE (BDE-206)	ND (12.9)	ND (13.2)	ND (11.4)	ND (3.95)	ND (0.63)	ND (0.85)	ND (10.2)
Nona-BDE (BDE-207)	ND (12.9)	ND (13.2)	ND (11.4)	ND (3.95)	ND (0.63)	ND (0.74)	ND (10.2)
Deca-BDE (BDE-209)	ND (64.5)	ND (65.9)	ND (57.1)	ND (80)	ND (3.16)	ND (3.66)	ND (71.3)
Total PBDE lower bound	25.2	0.0	43.9	4.8	12.5	5.5	0.0
Total PBDE middle bound	96.6	80.3	108.1	56.2	16.0	10.6	86.2
HBCD	ND (82)	ND (393)	ND (35)	ND (180)	ND (22)	ND (18)	300

Lower bound indicates ND estimated as 0; middle bound indicates ND estimated as one-half the LOD. Numbers in parentheses denote LODs.
